# Advances in Gene Therapy with Oncolytic Viruses and CAR-T Cells and Therapy-Related Groups

**DOI:** 10.3390/cimb47040268

**Published:** 2025-04-10

**Authors:** Yasunari Matsuzaka, Ryu Yashiro

**Affiliations:** 1 Division of Molecular and Medical Genetics, Center for Gene and Cell Therapy, The Institute of Medical Science, The University of Tokyo, Minato-ku, Tokyo 108-8639, Japan; 2 Administrative Section of Radiation Protection, National Institute of Neuroscience, National Center of Neurology and Psychiatry, Tokyo 187-8551, Japan; ryuy@niid.go.jp; 3 Department of Mycobacteriology, Leprosy Research Center, National Institute of Infectious Diseases, Tokyo 162-8640, Japan

**Keywords:** CAR-T, cytokine release syndrome, gene therapy, genome editing, oncolytic viruses, tumor microenvironment

## Abstract

Cancer gene therapy is attracting considerable attention as a new treatment method for overcoming intractable cancers. CAR-T cell therapy has already achieved remarkable results, particularly for hematological tumors. Because CAR-T cells can increase within the body, they have the advantage of requiring only a single administration. In addition, CAR-T cell therapy targeting the CD19 antigen has been established for relapsed or refractory disease in young people with CD19-positive acute B-cell leukemia (B-acute lymphoblastic leukemia, B-ALL) and diffuse large B-cell lymphoma (DLBCL). In addition to CAR-T cell therapy, oncolytic viruses represent a promising approach for cancer treatment, with some already in clinical use and others being researched for their potential benefits. These viruses infect and kill cancer cells, triggering an immune response that helps the body recognize and fight cancer. Oncolytic virus therapy is a form of immunotherapy that uses modified viruses to target and destroy tumor cells while potentially stimulating antitumor immune responses. These viruses have shown promising activity in clinical trials, with some approved for specific cancers like melanoma. Research is ongoing to improve their efficacy, expand their use to other cancer types, and overcome the logistical challenges associated with their delivery. Gene therapy can potentially treat diseases caused by recessive gene disorders like cystic fibrosis, hemophilia, muscular dystrophy, and sickle cell anemia, as well as acquired genetic diseases, such as cancer and viral infections like acquired immunodeficiency syndrome (AIDS).

## 1. Introduction

Gene therapy for cancer has a long history, with the idea of oncolytic virotherapy dating back to the early 20th century [1]. As early as the 1950s, cervical cancer was treated with live vaccine strains, the rabies vaccine, or wild-type adenovirus itself [2,3,4,5]. However, although chemotherapy has advanced rapidly since then, there have been occasional reports of the use of this treatment method [6]. During this time, however, research into viral proliferation, cancer, and biological defense mechanisms has progressed, driven by developments and technological innovations in molecular biology and genetic engineering [7,8]. Gene therapy for patients with cancer began in earnest in the USA, starting with the first administration of tumor necrosis factor (TNF) gene-transferred tumor-infiltrating lymphocytes to patients with malignant melanoma in 1989. Two-thirds of clinical research currently involves patients with cancer. Regarding oncolytic virotherapy, the efficacy of oncolytic viruses for human glioma has been reported [9,10,11,12,13]. Over time, oncolytic viruses based on various wild-type viruses have been developed, including adenovirus, measles virus, vesicular stomatitis virus (VSV), reovirus, HSV, and Sendai virus [13,14]. Adenoviruses are DNA viruses that can selectively replicate in and lyse tumor cells with defective p53 and Rb pathways [15]. Wild-type adenoviruses have been investigated as oncolytic agents, with some strains showing promising antitumor effects in clinical trials. Measles virus is a negative-sense RNA virus that can preferentially infect and kill cancer cells with high expression of CD46, a measles virus receptor [16]. Attenuated measles virus strains have demonstrated oncolytic activity against various cancers in preclinical and clinical studies [17]. VSV is a negative-sense RNA virus that can selectively replicate in interferon-deficient cancers. Wild-type and recombinant strains of VSV have demonstrated potent oncolytic effects against various tumor models [18]. HSV is a DNA virus that can be engineered to selectively replicate in and kill cancer cells. Both wild-type and engineered HSV strains have been studied as oncolytic agents, and some strains have been approved for clinical use [19]. Sendai virus is a negative-sense RNA virus that can replicate preferentially in cancer cells with defective interferon responses [20]. Numerous clinical trials using lytic viruses have been conducted, and products that have received regulatory approval have finally appeared (Table 1) [21,22,23,24].

The innovation and utility of gene therapy technology as a new modality with the new coronavirus vaccine have been widely recognized, and competition to develop gene therapy products is expected to become even more intense worldwide in the future [36,37].

Gene therapy involves the external introduction of therapeutic genes using gene transfer methods to replace missing genes or the delivery of cells with therapeutic functions using gene modification technology for control [38]. Viral vectors with high transduction efficiency are mainly used for gene transfer. Viruses with different properties are selected depending on the purpose of the treatment and the target cells. Immune therapies using recombinant proteins, such as monoclonal antibodies and other immune factors, have become an established part of cancer treatment in recent years, often used before or in combination with gene therapy approaches [39,40]. Immune checkpoint inhibitors, CAR-T cell therapies, and oncolytic viruses are examples of successful immunotherapies that have been integrated into cancer treatment. Gene therapy approaches to cancer, while showing promise, are still largely experimental and have not yet reached the same level of widespread clinical use as the traditional pillars or even some immunotherapies. A representative example of this is CAR-T cell (chimeric antigen receptor T cell) therapy, which has already achieved remarkable results, especially for hematological tumors [41,42,43,44,45,46,47,48,49,50,51,52,53,54,55,56,57,58,59,60,61,62,63,64,65,66,67,68,69]. In addition, oncolytic virus therapy, which uses a virus to selectively stop the proliferation of cancer cells and destroy them, is also considered a type of gene therapy because it uses recombinant viruses as gene therapy vectors [70]. It is attracting a lot of attention as a medical technology that has the potential to aid in the realization of radical treatment for solid cancers [71].

Research into treatments using genome editing technology is currently underway around the world [72,73]. This is because genome editing technology is being touted as an innovative medical technology that can treat diseases that were previously difficult to treat [74,75]. For example, more than half of difficult-to-treat diseases are caused by genetic mutations. Before the advent of genome editing technology, there was a technology to introduce normal genes from outside as a treatment method to make disease-causing genes work normally (called “gene therapy”). However, gene therapy leaves the abnormal gene behind, and the introduced gene is randomly integrated into the genome, so there are limitations, such as the risk of damaging other normal genes and the inability to regulate the expression of the introduced genes.

Therefore, there are still many safety issues, and the technology is still in its developmental stage. This review focuses on gene therapy and provides an overview of marketed products, development pipeline trends, technology development trends, and various development issues, such as safety, manufacturing, quality control, regulatory aspects, and so on. In this review, we have focused on new areas of gene therapy, including CAR-T-, AAV (adeno-associated virus)-, or HSV (herpes simplex virus)-based oncolytic viruses. In addition, while the five-year survival rate for all cancers at all sites and all clinical stages has gradually improved to approximately 68.4%, the prognosis for intractable cancers such as pancreatic cancer, biliary tract cancer, malignant mesothelioma, small-cell lung cancer, and ovarian cancer remains poor. Pancreatic cancer, for example, has an extremely poor prognosis among malignant tumors, with a five-year survival rate of approximately 9%.

## 2. Tumor Microenvironment (TME) and Gene Therapy

Cancer gene therapy is attracting a lot of attention as a new treatment method to overcome intractable cancers [76]. Oncolytic viruses are naturally occurring or genetically engineered viruses that selectively replicate and spread within tumor tissue, destroying it without causing undue damage to normal tissue [71,77]. Oncolytic virus therapy using these viruses is classified as in vivo gene therapy, the direct introduction of genes into the body for cancer [70,78,79]. Oncolytic viruses can remodel the TME and stimulate antitumor immune responses through various mechanisms. In interactions with immune cells, oncolytic viruses can enhance the infiltration and activation of immune cells such as natural killer (NK) cells, CD8+ T cells, and dendritic cells (DCs) within the TME [80,81]. They reduce immunosuppressive cells such as regulatory T cells and myeloid-derived suppressor cells, thereby overcoming immune suppression in the TME [81,82]. Oncolytic virus infection causes the immunogenic cell death of tumor cells, releasing tumor-associated antigens and danger signals that activate DCs and prime antitumor T cell responses [80,82]. In the expression of immunomodulatory molecules, oncolytic viruses can be engineered to express immunostimulatory cytokines, chemokines, or tumor antigens that further enhance antitumor immunity [80,82,83]. By overcoming immunosuppressive pathways, oncolytic viruses can counteract immunosuppressive mechanisms such as the indoleamine 2,3-dioxygenase (IDO)–kynurenine–aryl hydrocarbon receptor (AhR) pathway in the TME, allowing for better immune cell activation [84]. The following are crucial for successful oncolytic virotherapy: (i) the efficient delivery of oncolytic viruses to the TM, (ii) the robust replication and spread of oncolytic viruses within the tumor bed, causing oncolysis and the release of immunogenic signals [80,84], and (iii) the activation of potent innate and adaptive antitumor immune responses by the oncolytic virus, acting as an in situ immunotherapeutic agent [80,81,82]. Thus, OVs can transform the immunosuppressive TME into an immunostimulatory environment through direct oncolysis, the expression of immunomodulatory molecules, and interactions with various immune cell populations, leading to potent antitumor immunity [80,81,82,83,84].

**Figure 1:**
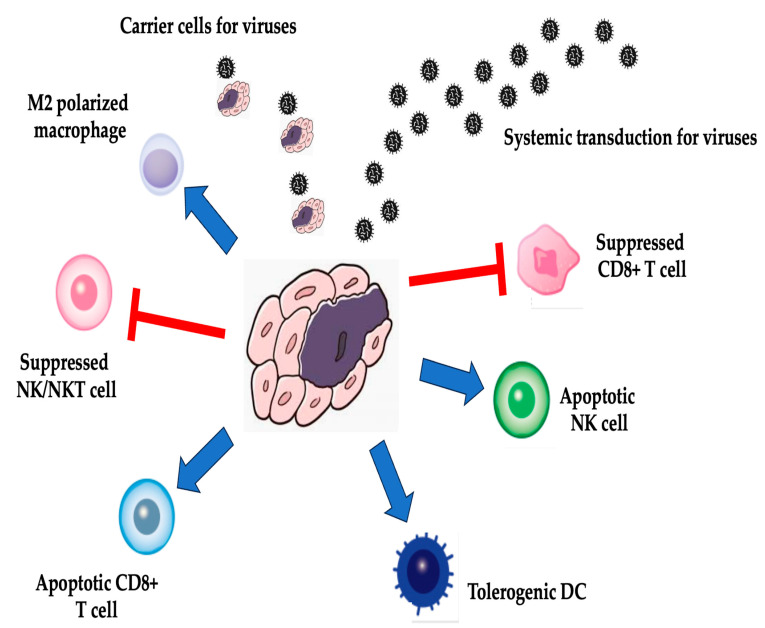
The mechanism of action of oncolytic viruses in the tumor microenvironment. Tumor microenvironment: interactions between macrophages, NK cells, CD8+ cells, and DCs. CAR-T cell therapy involves reprogramming T cells to express chimeric antigen receptors (CARs) that target specific tumor antigens, leading to high response rates in cancer treatment. The oncolytic virus must be delivered successfully to the TME. The oncolytic virus must replicate and spread efficiently in the tumor bed, causing oncolysis and the release of tumor-selective immune-stimulatory molecules. The oncolytic virus must function as an immunotherapeutic agent to activate potent innate and adaptive antitumor immune responses. Oncolytic viruses can repolarize tumor-associated macrophages from an immunosuppressive M2 phenotype to a pro-inflammatory M1 phenotype, enhancing antitumor immunity. Oncolytic virus infection can activate NK cells, contributing to antiviral and antitumor responses. Oncolytic viruses promote the infiltration and activation of cytotoxic CD8+ T cells, enhancing tumor cell killing. Oncolytic virus-mediated cell lysis releases TAAs, which are taken up by DCs for antigen presentation, stimulating adaptive immune responses. Combining oncolytic viruses and CAR-T cells can potentially enhance CAR-T cell efficacy in solid tumors by increasing tumor penetrance and overcoming the immunosuppressive TME.

Many cancer cells have impaired defenses against viral infection, making them much more susceptible to infection by most viruses than normal cells [85,86,87]. When oncolytic viruses are taken up by cancer cells, they replicate efficiently, lysing and killing the host cancer cells [88,89]. The tumor microenvironment influences some immune cells [72,90,91]. Activated cytotoxic T cells stimulated by these cells then infiltrate the tumor and destroy cancer cells. The TME is a complex system where different immune cells interact to influence tumor growth and immune responses (Figure 1). The following are the key findings from search results regarding the interactions between macrophages, NK cells, CD8 T cells, and DCs in the TME: (1) NK cells and macrophages—NK cells can regulate macrophage phenotype and influence antitumor immune responses. The activation of NK cells by environmental stimuli can promote the polarization of myeloid cell subsets toward a pro-inflammatory state, thereby enhancing antitumor responses [92]. (2) DCs and CD8 T cell immunity—DCs are critical in regulating the balance between CD8 T cell immunity and tolerance to tumor antigens. DCs play a central role in the activation of CD8 T cells through the cross-presentation of antigens, but TME-mediated suppression can lead to T cell dysfunction. (3) Macrophages in the TME—Macrophages form a spatial barrier in the TME that can attenuate the tumor-killing function of CD8+ T cells. The aggregation of macrophages in the peripheral stroma of tumors inhibits tumor immunity and promotes tumor progression [93]. (4) DC subsets—Different subsets of DCs play distinct roles in cancer immunity. For example, cDC1s are associated with increased T cell infiltration and improved outcomes, highlighting their positive role in generating antitumor immunity [94]. (5) T cells, NK cells, and macrophages in immunotherapy—T cells, NK cells, and macrophages are essential in cancer immunotherapy. T cells have high antitumor specificity, while NK cells have strong cytotoxic effects on tumor cells. Understanding their interactions is critical for the development of effective immunotherapies [95]. These findings underscore the intricate interplay between immune cells within the TME and their impact on antitumor immune responses. Manipulating these interactions may hold promise for improving immunotherapies and outcomes for cancer patients.

Various studies have explored different viral vectors for reprogramming T cells to express CARs, such as lentiviral vectors and adeno-associated viral vectors, to increase specificity and efficacy while minimizing off-target effects [96]. The in situ generation of CAR-T cells using viral vectors has shown promising results in preclinical studies and has the potential to simplify the manufacturing process and reduce costs associated with the ex vivo production of CAR-T cells [96]. However, challenges such as the induction of immune responses by foreign antigens and integration near active genes need to be addressed before the widespread clinical application of in situ-generated CAR-T cells [96]. Overall, CAR-T cell therapy represents a breakthrough approach in cancer immunotherapy, offering personalized treatment options by reprogramming patients’ immune cells to effectively target and destroy cancer cells. Ongoing research focuses on improving CAR-T cell therapies for solid tumors by developing “armored” CAR-T cells that can navigate immunosuppressive tumor environments or target single surface antigens on cancer cells. Despite obstacles such as tumor heterogeneity and off-tumor toxicity, advances in CAR-T technology continue to revolutionize cancer treatment, making it a standard of care with significant potential for further development and refinement.

## 3. HSV-Based Oncolytic Viruses

There are HSV-based oncolytic viruses, and the advantages of using HSV are that (i) it has a large capacity to carry different therapeutic genes, (ii) it has strong antitumor activity, and (iii) unlike conventional anticancer drugs, oncolytic viruses are not toxic to normal cells and cause almost no side effects even if an adverse event occurs [97,98,99,100]. Safety is assured because an antiviral drug, acyclovir, already exists for HSV. HSVs, including HSV-1 and HSV-2, have relatively large double-stranded DNA (dsDNA) genomes, ranging from 130 to 230 kilobase pairs (kbp) in size. The diameter of HSV-1 virions without the envelope glycoprotein spikes is approximately 186 nm, while the diameter including the spikes can increase to 225 nm. In comparison, the diameters of virions from other virus families can vary considerably, from about 20 to 300 nm. Thus, while it is true that herpesviruses such as HSV carry large dsDNA genomes, the size of their virions is not necessarily the same as that of all other types of viruses. Herpesvirus virions are relatively large compared to some other types of viruses. HSV is characterized by its ability to infect almost all cancer cell types and its strong oncolytic effect [101,102,103]. Specifically, in terms of first-generation HSV, a gene is mutated at one site to selectively replicate in tumors. Second-generation HSV increases safety by modifying genes at multiple sites. For third-generation HSV, the introduction of foreign genes necessary is for treatment to enhance antitumor effects [104,105,106]. For malignant melanoma, the second-generation oncolytic HSV Imlygic (talimogene laherparepvec; T-Vec) was approved as a first-in-class drug in the United States and subsequently in Europe. T-Vec not only has a direct antitumor effect due to the cancer cell-specific proliferation of the virus but also enhances antitumor immunity by carrying the granulocyte-macrophage colony-stimulating factor (GM-CSF) gene, which has an immunostimulatory effect [107,108,109,110]. The third-generation oncolytic HSV Deritact (teserpaturev; also known as G47Δ) has three HSV genes modified to enhance its ability to replicate in cancer cells and its antitumor immunity [97,101,111]. There is also T-hIL12, which has G47Δ and has been loaded with the IL12 gene, which has a strong ability to induce immune cells, to further enhance antitumor immunity [112,113,114]. In addition to C-REV, a naturally mutated oncolytic HSV, there are numerous options to improve the efficacy of treatment, including the development of oncolytic viruses engineered using genetic modification technology to express immunomodulatory proteins in cancer cells, research on ways to escape from naturalizing antibodies, and the verification of the optimal regimen, including combinations with other drugs, etc. [78,115].

## 4. CAR-T Cell Therapy and Immune Effector Cells

**Figure 2:**
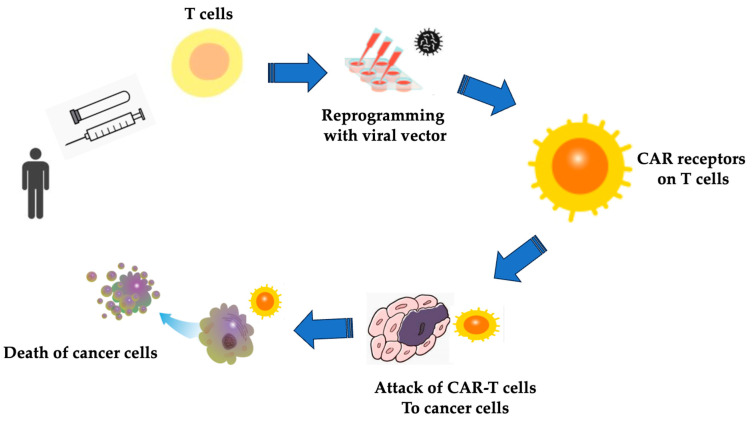
CAR-T cell therapy. CAR-T cell therapy involves reprogramming T cells to express chimeric antigen receptors (CARs) that target specific tumor antigens, leading to high response rates in cancer treatment. It is a type of treatment in which a patient’s T cells are engineered in the laboratory to bind to and kill cancer cells. The gene for a special receptor, CAR, is inserted into the T cells in the laboratory. Millions of CAR T cells are grown in the lab and then fused and given to the patient. The CAR-T cells can bind to an antigen on the cancer cells and kill them. The process of CAR-T cell therapy. (I) T cell collection: the patient’s T cells are extracted from their blood through a process similar to blood donation. (II) Genetic modification: the collected T cells are genetically engineered in a laboratory to express CARs designed to target specific cancer antigens. (III) Expansion: the modified CAR-T cells are multiplied in large numbers to create a potent anticancer force. (IV) Preparatory treatment: the patient may undergo lymphodepletion to reduce competing immune cells and enhance CAR-T cell efficacy. (V) Infusion: the engineered CAR-T cells are infused back into the patient’s bloodstream. (VI) Monitoring: after infusion, patients are closely monitored for treatment response and potential side effects.

Until recently, no treatment could significantly alter the prognosis of patients with hematopoietic tumors resistant to chemotherapy [116,117,118,119]. However, this is changing with the advent of chimeric antigen receptor (CAR)-delivered T cells, which have fundamentally different antitumor mechanisms (Figure 2). CAR-T cell therapy for targeting the CD19 antigen has been established for relapsed or refractory disease in young people with CD19-positive acute B-cell leukemia (B-acute lymphoblastic leukemia, B-ALL) and diffuse large B-cell lymphoma (DLBCL) [120]. A treatment approach that involves the culture, expansion, and administration of immune effector cells outside the body and administers them is called adoptive immune cell therapy [121]. To date, interleukin (IL)-2-activated NK cell therapy, tumor-infiltrating lymphocyte (TIL) therapy that infiltrates cancer tissue, and virus-specific T cells have been tested and shown some efficacy [122,123]. However, it is not easy to efficiently generate antigen-specific T cells in a short time. However, when tumor antigens are identified, those with high antigenicity can be found. T cell clones and antibodies that recognize them can be generated, and the T cell receptor (TCR) and antibody genes can be inserted into a vector. When the genes are introduced into T cells, the desired antigen-specific T cells can be generated in a short time [124,125,126]. Antibody therapy requires repeated administration. However, in the case of CAR-T cells, which have the specificity of antibodies, because they can proliferate in the body, they have the advantage of requiring only a single administration [127]. In addition, NK cells and other cells are necessary to achieve antibody-dependent cytotoxic activity, but chemotherapy can reduce the number of these cells and may be less effective [128]. However, CAR-T cells themselves have cytotoxic properties and can be expanded outside the body and administered to the site of need [116,129,130,131,132]. In addition, non-CAR genetic modifications can be added, cytokine genes can also be integrated to enhance function, and chemokines and their receptors can be expressed to promote the recruitment of other immune cells.

Although it is difficult to determine exactly what the prototype of CAR-T cells is, it is possible to express antibody molecules, which are antigen receptors used by B cells, on T cells [133,134]. The variable regions of the heavy and light chains of antibodies and the constant region of the TCR have been combined and expressed in the mouse T cell line EL4, and EL4 has been shown to be activated by antigens. Other features of CAR-T cell therapy include the following: (i) it is universal with no MHC/HLA restriction, (ii) T cells obtained from anyone can be modified by gene transfer, and (iii) T cells can proliferate and exert their functions. The currently used CAR is a single chain consisting of a light chain and a heavy chain linked in tandem [135,136,137]. The single strand is an important component because if the genes were introduced into each part separately, the expression levels would be different, or the genes would bind to different molecules [138]. Previously, the only intracellular domain that transmitted antigen signals into cells and activated T cells was the intracellular domain of the CD3ζ chain, a signal transduction molecule of the TCR complex [139]. However, the short-term in vivo survival of CAR-T cells, which consist of single-chain antibodies against the B-cell antigen CD20 and CD3ζ chains, has become a problem [135,140,141,142]. Normally, T cell activation requires signals from costimulatory molecules such as Cd28 and Cd137(4-1BB) in addition to signals from the TCR. In particular, the latter increases the degree of T cell activation and affects survival in the body and the generation of memory T cells. One report was the first to introduce a method for tandemly integrating the intracellular domain of this costimulatory molecule into the intracellular domain of the CAR [143,144]. Subsequently, the use of domains derived from various costimulatory molecules, including CD137, has been attempted.

However, the current mainstream method is to combine CD28 or CD137 with CD3ζ [145,146]. For convenience, the CAR of CD3ζ alone is called the first generation, those that add one costimulatory molecule to CD3ζ are called the second generation, and those that add two are called the third generation [146,147,148]. In addition to cytotoxic activity, significant improvements in proliferation ability, cytokine production ability, and in vivo survival rate have been observed since the second generation [149,150,151]. For example, depending on the size of the molecule on the cell surface targeted by the CAR and the location of its epitope at the top of the molecule near the cell membrane, it may be necessary to optimize the length of the “hinge” portion of the extracellular CAR [115,144,144]. CAR-T cells are T cells taken from patients with cancer into which a CAR has been introduced.

By re-administering CAR-T cells, which have been cultured in vitro for about two weeks, to patients with cancer, CAR-T cell therapy, which recognizes cancer antigens and targets them for attack and treatment, is classified as ex vivo gene therapy, in which genes are introduced into and administered to cells removed from the body [152,153,154]. The CAR is composed of three parts in the intracellular region, which are an extracellular region consisting of an antigen-binding domain and a hinge domain that specifically binds to target molecules, a transmembrane region that attaches the CAR to the cell membrane and transmits the ligand recognition signal into the cell, and costimulatory and signaling domains that directly contribute to T cell activation. Fifth-generation CAR-T cells are currently under development [146,149,155,156]. Specifically, first-generation CAR-T cells are T cells in which CD3ζ is directly linked to a single-chain antigen antibody scFv and expressed as a single protein [135,157]. In second-generation CAR-T cells, a costimulatory domain, CD28 or 4-1BB, is linked between the first-generation CAR-T scFv and CD3ζ [117,158]. Third-generation CAR-T cells involve the binding of two costimulatory domains (CD28 and 4-1BB) [157,159,160,161].

Furthermore, when the antibody-derived constant region is used directly as a hinge, it activates NK cells and influences the survival of CAR-T cells [117,118,134,162]. In addition to the structure, the introduction of pretreatments has also contributed to the dramatic improvement in the efficacy of CAR-T cell therapy [163]. The reason for the poor survival of infused T cells after in vitro expansion was thought to be competition with the patient’s natural T cells [117,164]. When the number of lymphocytes decreased due to lymphocyte depletion conditions, cytokines such as IL-7 and IL-15 were secreted, which allowed the infused genetically modified T cells to rapidly proliferate in the body [165,166]. Fludarabine and cyclophosphamide are commonly used for treatment. However, melphalan and bendamustine, which have strong effects on lymphocytes, and sometimes low-volume whole-body irradiation have also been used in combination. In addition, the duration of the in vitro culture period and the degree of differentiation of the T cells are also factors related to T cell survival after transfusion [167]. In the early stages of development, long-term culture was performed in the hope that it would be more effective if the cells were grown in large quantities outside the body, reaching a level of 10 billion cells. However, once infused, the cells quickly disappear from the patient’s peripheral blood. This is because the surface characteristics of T cells change from central memory to differentiated effector memory and then to terminal effector memory [168]. CAR-T cells that retain memory characteristics can activate and proliferate when they encounter antigen-expressing tumor cells in the body, so the culture period is now being kept to a minimum [117,169,170].

CAR-T cell therapy is a type of adoptive immunotherapy using genetically modified T cells [171]. In recent years, it has attracted much attention as a new treatment for B-cell tumors such as acute lymphoblastic leukemia, malignant lymphoma, and chronic lymphocytic leukemia, as well as multiple myeloma. CAR-T cells targeting the CD19 antigen have shown a high rate of remission induction in refractory B-cell tumors [172,173]. CAR-T cells can be prepared in a short time, and the use of antibodies as antigen receptors, which have a higher affinity than the T cell receptors of T cells, has contributed to their success [134,174]. In addition, the use of antibodies as antigen receptors eliminates the need to consider the HLA restriction of T cells. The structure of a CAR consists of the Fab portion of the antibody as the extracellular domain and the T cell signaling domain as the intracellular domain [144,175]. Various modifications have been made to the intracellular domain to activate T cells, enhance their functions, and confer long-term survival in the body [117,176,177]. However, serious side effects such as cytokine release syndrome can occur frequently after treatment, and some relapses have been observed in the long term; therefore, there are still issues that need to be resolved in the future [178].

Next-generation (fourth and fifth generation and beyond) CAR-T cell therapies involve the modulation of CAR activity with small molecules to increase safety (switch CAR-T cells), to increase therapeutic efficacy by expressing IL-7 and CCL9, etc. (prime CAR-T cells), with T cells obtained directly from a bank rather than derived from patients to save time and costs (universal CAR-T) [149,179,180,181]. These are generated by expressing a CAR containing an scFv that recognizes CD19, a surface antigen of B cells, or autologous T cells [173,182]. Next, the search for effective target antigens other than CD19 has progressed by targeting the B-cell maturation antigen (BCMA), which is expressed only on plasma cells and myeloma cells of patients with multiple myeloma [183,184,185]. In addition to CAR-T cell therapy, there is a treatment method mainly for solid tumors using TCR-T cells, in which the T cell receptor (TCR) gene, which specifically recognizes tumor antigen peptides, is introduced into T cells using a viral vector [186,187].

Cytokine release syndrome (CRS) is a syndrome that occurs when the immune system is overactivated [188,189,190]. In most cases, it occurs from the day after administration to day 14 (median around day 3 for CD19 CAR-T cells) [117,134]. The disease progresses rapidly, and in severe cases, respiratory and circulatory management is required, and death may occur. Symptoms and findings may be similar to those of hemophagocytic syndrome. CRS is not limited to CAR-T cell therapy but is now thought to be caused by the activation of immune effector cells, including T cells [191,192]. This includes cases where immune cells are activated by blinatumomab or antibody therapy [193]. Neurologic symptoms after CAR-T cell therapy may also occur due to coexisting liver injury, electrolyte abnormalities, adverse effects of administered drugs, and abnormal hypertension [194,195]. These symptoms should be excluded from the diagnosis of what was initially named CAR-related encephalopathy syndrome (CRES) [196]. However, other immune effector cells may also be responsible, and because of this potential, the name immune effector cell-associated neurotoxicity syndrome (ICANS) has been proposed [197]. Although the cause is not yet clear, it is distinct from CRS because it often develops after CRS sedation and the symptoms are different [198,199]. In addition to T cell overactivation, inflammatory cytokines in the cerebrospinal fluid and blood–brain barrier abnormalities may be responsible, and indeed, CAR-T cells have been detected in the cerebrospinal fluid [200,201,202,203,204]. Initially, symptoms included headache, hallucinations, tremor, and myoclonus, but these were not used alone for diagnosis due to a lack of specificity [205]. Currently, encephalopathy, impaired consciousness, seizures, hemiparesis/paraplegia, and cerebral edema on imaging are the main symptoms [206,207,208]. Aphasia is a symptom of encephalopathy, and symptoms such as disorientation, dysgraphia, and anomia are seen from the onset [209].

Because CAR-T cells target a single antigen, CAR-T cells are not expected to be effective if a variant loses its antigenicity [117,118,134,210]. The loss of the CD19 antigen occurs at a frequency of 10 to 20% in pediatric B-ALL [117,211]. This includes deletions of the CD19-containing gene locus, novel CD19 frameshift mutations, and mutant mRNAs that skip exon 2, which encodes the epitope of the antibody used in the CAR. A similar loss of the antigen occurs in CD22 CAR-T cells. These findings suggest that even after CAR-T cell therapy, immunoediting occurs and immune-resistant clones are selected [212,213,214]. To overcome the problem of antigen loss variants, dual CAR-T or tandem CAR-T cells, which have two types of antibody binding sites, have been developed [215,216,217,218,219]. In addition, the use of CAR-T cells has made it possible to damage tumor cells expressing low levels of antigens that cannot be damaged by antibody therapy, while normal cells can be damaged if they express the same antigen, albeit weakly [117]. After CD19 CAR-T cell therapy, normal B lymphocytes also disappear, resulting in low levels of immunoglobulin that need to be replaced, known as on-target off-tumor reactions [215,220,221]. In addition, antibodies can also cause unexpected cross-reactivity as off-target reactions, so care must be taken when selecting therapeutic targets. In addition, the patient’s immune response to the antibody’s variable region and vector-derived proteins used in CAR-T cells can lead to the rejection and elimination of CAR-T cells [134,210]. In addition, in solid tumors, the tumor microenvironment is extremely immunosuppressive, so the expected effects are often not achieved, and combination therapy is considered [222,223]. Conventional CAR-T cells are customized for each patient and require a large amount of money, including the costs of peripheral blood collection and transportation, the use of a sterile culture processing facility, viral vector gene transfer, quality testing, and transportation to a treatment facility [224,225,226]. However, suppose that CAR-T cell therapy alone can provide long-term survival for many patients. In this case, the costs may not be very different from those required for repeated chemotherapy or allogeneic hematopoietic stem cell transplantation for B-ALL [224]. For example, when CAR-T cells are used in patients at high risk of relapse with minimal residual disease, outcomes may improve, and costs may decrease if allogeneic hematopoietic stem cell transplantation becomes unnecessary [227,228,229].

## 5. The Therapeutic Effect of Ex Vivo or In Vivo Therapy and Issues

With regard to oncolytic virus therapy and CAR-T cell therapy, unfortunately, at present, the therapeutic effect of single therapy is still limited, especially in solid tumors [230,231]. Contributing factors include the presence of a dense fibrotic stroma, which acts as a barrier to drug delivery in refractory cancers, and the immunosuppressive TME [232,233,234,235,236]. Fortunately, however, these gene therapies are highly compatible. It is expected that the aforementioned problems can be overcome by combination therapy. For example, through the use of mesenchymal stem cells (MSCs) as carriers for oncolytic viruses, it is possible to efficiently deliver them to metastatic sites and improve antitumor effects [80,232,233,234,235,236,237,238,239,240]. TME remodeling induced by oncolytic virus infection improves the infiltration, persistence, and functional limitations of CAR-T cells, resulting in higher therapeutic efficacy [117,241,242]. In addition, MEKi + STAT3i administration to pancreatic cancer remodeled the TME and improved the antitumor effect of immune checkpoint inhibitors [243]. In addition, oncolytic viruses also synergistically enhance the therapeutic effects of immune checkpoint inhibitors [244,245,246,247,248].

The number of development pipelines for gene cell therapy and in vivo gene therapy has increased exponentially since the 2010s. CART-T cells are becoming established as a modality that can be expected to have reliable antitumor effects [214,249,250,251,252]. Regarding in vivo gene therapy, a relatively large number of pipelines had existed since the 1990s, but the number of pipelines stagnated for a while in the 2000s [253]. Around 2010, the number of pipelines increased steadily, triggered by successive demonstrations of clinical efficacy for Parkinson’s disease, aromatic amino acid decarboxylase (AADC) deficiency, Leber’s congenital amaurosis, hemophilia, spinal muscular atrophy, etc. [254]. In gene cell therapy, about 50% of treatments in the pipeline come from the US and about 26% from China, and these two countries are unique in that they account for about 3/4 of the total [255,256]. China has many antitumor drugs in the pipeline, including various CAR-T cells and CAR-NK cells [257]. For in vivo gene therapy, about 57% of treatments in the pipeline come from the US, followed by European countries such as France, the United Kingdom, and the Netherlands [258]. For oncolytic viruses, the total number of products is still small, with approximately 42% of products in the pipeline coming from the US, followed by China with 11% and Canada with 9% [44,259].

On the other hand, several challenges remain in advancing research and development in gene therapy [260,261]. From the perspective of enriching basic research, it is necessary to refine modalities to further improve efficacy and safety and to promote the industrialization of gene therapy [260]. In the United Kingdom, the “Catapult Concept” was proposed in 2010. A nationally led project is being promoted to establish industry–academia collaborative centers (catapults) for each major area that will drive future UK economic growth, with intensive budget investment. As part of this, the Cell Therapy Catapult (CGT Catapult) was established in 2018 as an extension of the Cell Therapy Catapult, which was established in 2012 and plays a central role in research and development in the field. The CGT Catapult’s vision and mission are fourfold: (i) to achieve innovation through the creation of industry–academia networks, (ii) to commercialize innovation, (iii) to complement industry–academia collaboration with unique research and manufacturing facilities and expertise, and (iv) to facilitate the growth of the UK ecosystem through collaboration with industry, research institutions, government, health and medical institutions, trade associations, and international organizations.

Not only does gene therapy, as a new technology, face particularly high uncertainties in research and development, but the lack of experienced and specialized human resources and the high manufacturing costs required from the early stages of research and development are high barriers to entry, becoming an impediment to commercialization as an industry [260,261]. The CGT Catapult strongly recognizes these points. In addition to creating a system that allows for the flexible sharing of manufacturing facilities, including the production of investigational drugs, according to the needs of users, it intends to develop cell therapy and gene therapy into a major industry in the UK in the future by employing and training many people, thereby establishing it as a place to develop human resources [262]. Notable initiatives in the U.S. include PaVe-GT (Platform Vector Gene Therapy) and the BGTC (Bespoke Gene Therapy Consortium). PaVe-GT is an NCATS-led project within the NIH. The project will conduct research and development in gene therapy for four different rare diseases: two types of organic acidemia (PCCA deficiency and MMAB deficiency) and two types of congenital myasthenic syndrome (DOK7 deficiency and COLQ deficiency). In this project, the purpose is to verify the possibility of streamlining gene therapy research and development through the use of standardized methods, such as the creation of candidate formulations using a common capsid and common manufacturing and purification methods, the adoption of common nonclinical testing and CMC evaluation methods, and the efficient conduct of clinical trials through master protocols, etc. [263]. The BGTC is a consortium involving a total of 27 different stakeholders, including several NIH research institutes such as the NCATS, the FDA, 10 pharmaceutical companies, and patient organizations. In addition to the establishment of rational and effective standard assessment items and assessment methods, the status of regulations and uniform manufacturing processes will also be considered. The BGTC will also explore ways to reduce the cost of research and development in gene therapies for rare diseases, paving the way for commercial viability and sustainability [264].

For example, in gene therapy, issues such as the limitations of animal models in safety assessment and quality assurance, including various impurities and empty capsids, remain [265]. In terms of the need to advance and enrich basic research, these remain global challenges. Therefore, further scientific and technological breakthroughs are needed to refine gene therapy into more precise modalities for medical applications [266]. Specifically, the following issues need to be solved: (i) improving gene expression efficiency and reducing dosage, (ii) controlling gene expression and optimizing the expression amount and expression time, (iii) elucidating toxicity mechanisms and establishing toxicity avoidance methods, (iv) adding targeting tropisms for a systemic type of action and tissue-selective delivery, (v) establishing high-quality manufacturing methods, including cell culture methods, and (vi) significantly reducing the manufacturing cost of drugs needed for treatment by comprehensively solving these issues, among others [267]. If these hurdles can be overcome, it may also lead to gaining superiority over other modalities by replacing them with gene therapy that can be treated with a single administration [268]. Regarding the importance of gene therapy for diseases based on genetic mutations, unlike conventional drugs that can be widely used by heterogeneous groups of patients, it is assumed that therapeutic drugs will not be developed for this group of patients, which could lead to drug loss, which is more serious than drug delay. In addition, gene therapy technology can be applied not only to rare diseases but also to various other diseases [266]. If research is only conducted on rare diseases, there is a possibility that profits will not be commensurate with research and development investment, which carries a high risk of failure [269]. However, in research and development in orphan drugs for rare diseases, it is possible to keep the scale of manufacturing and clinical trials relatively small [270]. It would be reasonable and effective to scale up or intensify the production of therapeutic drugs for a larger patient population based on the outcomes of this series of research and development efforts [271,272]. Most importantly, “this technology can also be applied to the development of vaccines for emerging infectious diseases” [273,274].

## 6. Aspects of Both CAR-T Cell Therapy and Oncovirotherapy

CAR-T cell therapy and oncovirotherapy are innovative approaches to cancer treatment that hold great promise for overcoming the challenges posed by solid tumors [78]. The combination of CAR-T cells and oncolytic viruses has the following benefits: (1) Enhancing tumor-specific T cell functions—viruses can be engineered to selectively deliver therapeutic genes to the tumor microenvironment, potentially enhancing the effector functions of tumor-specific T cells. (2) Overcoming limitations in solid tumors—While CAR-T cells have shown remarkable success in hematological malignancies, their efficacy in solid tumors has been limited. Combining oncolytic viruses with CAR-T cells represents a strategy to enhance viral delivery to tumors and improve oncolysis, creating a positive feedback loop. In terms of challenges and future directions, identifying the most appropriate oncolytic virus for combination with CAR-T cells remains a challenge. Strategies to increase tumor immunogenicity and reduce the immunodominance of viral antigens are critical to optimize this combined therapy.

Clinical status: (1) Curative potential—Preclinical studies have demonstrated the curative potential of CAR-T cells and oncolytic viruses. However, as single agents, their efficacy is limited to specific patient subsets, necessitating combinations with other targeted therapies for more efficient and durable responses against heterogeneous tumors and their microenvironment [78]. (2) Combinational approaches—Ongoing clinical evaluations focus on combining CAR-T cells and oncolytic virotherapy with other therapies to improve cancer treatment outcomes. Further investigations tailored to each tumor type are essential to improve the antitumor effects of CAR-T cells [78]. The side effects of CAR-T cell therapy include the following: (1) Weakened immune system—patients undergoing CAR-T cell therapy may experience a weakened immune system, leading to an increased risk of serious infections. (2) Low blood cell counts—this therapy may also result in low blood cell counts, which can increase susceptibility to infection, fatigue, bruising, or bleeding. The prompt reporting of side effects is critical for timely treatment.

## 7. Ex Vivo and In Vivo Gene Editing for Hereditary Diseases

Throughout the history of gene therapy research and development, safety has been an issue. The advent of hematopoietic stem cell gene therapy for X-linked severe combined immunodeficiency disease (X-SCID), which occurred in 1999, was the first time in the world that gene therapy alone had clear medical efficacy [275]. However, it was subsequently reported that a number of leukemias developed in treated patients, which had a serious impact on research and development in gene therapy in general. Subsequent research elucidated the mechanism of toxicity, and it became clear that the retroviral vector used at the time increased the risk of carcinogenicity by inserting genes at unintended sites. In recent years, improved retroviral vectors have been developed, and this risk has been significantly reduced. In addition, in recent years, in vivo gene therapy using adeno-associated virus (AAV) vectors has been actively researched and developed [257,276,277,278,279]. AAV vectors have been widely used as vectors for in vivo gene therapy because they are derived from a non-pathogenic virus, have high safety, show relatively high gene transfer efficiency even in non-dividing cells, have multiple serotypes with different tissue tropisms, and can target different organs in the body [257,280]. However, AAV has also shown some toxicity in clinical trials, and safety issues remain [257,281,282,283]. In addition to the risk of oncogenicity, serious side effects recognized in clinical trials include hepatotoxicity, thrombotic microangiopathy (TMA), and neurotoxicity [284]. Although various hypotheses have been proposed regarding the mechanisms of this toxicity, no clear conclusions have been reached yet.

## 8. Gene Therapy for Hereditary Disease

On the other hand, it is now possible to delete only abnormal genes or insert normal genes into the target genome, thus enabling treatments that were not possible with conventional gene therapy, such as precisely repairing genetic mutations. Thus, conventional gene therapy is a treatment method that uses a technology that “integrates” only a specific gene, whereas treatment using genome editing technology normalizes abnormal disease-causing genes by “editing” only specific genes. There are two types of treatments using genome editing technology: (i) a method in which after cells have been taken from patients (sometimes, cells from other people are used) and the problematic gene has been modified by genome editing “ex vivo”, outside the body, cells that have become normal are returned to the body, and (ii) a method of performing genome editing “in vivo”, inside a patient’s body [47]. Treatments using ex vivo genome editing use cells taken from outside the body and are therefore classified under the therapeutic umbrella of “cell therapy” [285]. On the other hand, treatment using in vivo genome editing is included as a treatment method in “gene therapy” [286,287,288,289,290]. In addition, ex vivo genome editing has the advantage of being technically safer than in vivo genome editing because it uses cells taken from outside the living body and it is possible to select cells that have undergone precise genome editing [285,291,292]. However, there are only a limited number of diseases that can be treated with ex vivo genome editing therapy [293,294]. Depending on the type of disease, such as diseases that will not improve unless the diseased tissue itself is treated, gene therapy using in vivo genome editing technology, which can directly restore the diseased area to normality, may be appropriate [287,295]. Thus, treatments using genome editing technology are expected to make it possible to treat diseases for which there is no cure [296].

In recent years, drug discovery modalities have diversified. In addition to small-molecule drugs, various modalities such as recombinant proteins, antibody drugs, gene therapy, and cell therapy are beginning to be put into practical use as pharmaceuticals [297]. Cases where foreign genes are introduced, e.g., DNA vaccines, mRNA vaccines, etc., may not be defined as gene therapy [298]. Gene therapy and vaccines are classified as separate categories, even though they are drugs that use similar viral vectors to introduce genes [299]. In addition, when the elementary technologies of gene therapy are analyzed, they are divided into three categories: “in vivo gene therapy”, “ex vivo gene therapy”, and “oncolytic virus”.

CRISPR-Cas9 is a powerful tool for efficient viral vector generation [300,301,302,303]. Gene therapy for inherited hematological diseases using CRISPR/Cas9 technology has been approved in both the United Kingdom and the United States. In the UK, the Medicines and Healthcare Products Regulatory Agency (MHRA) was the first in the world to approve a breakthrough gene therapy called Casgevy for the treatment of sickle cell disease and beta-thalassemia. Casgevy works by editing the faulty gene in a patient’s bone marrow stem cells, allowing the body to produce functional hemoglobin. The treatment involves removing stem cells from a patient’s bone marrow, editing them in a laboratory using CRISPR gene editing technology, and then infusing them back into the patient. Clinical trials have shown promising results, with patients experiencing the relief of symptoms and a reduced need for blood transfusions. The U.S. Food and Drug Administration is also considering approval for Casgevy, known as Exa-Cel, for sickle cell disease.

Traditional gene therapies do not always aim to integrate the therapeutic gene into the host genome. Many gene therapy approaches use episomal vectors that remain extrachromosomal without integrating into the chromosomes of the host cell [304,305]. Episomal vectors are non-integrating, circular DNA molecules that replicate independently of the host genome. They offer several advantages over integrating viral vectors, including a reduced risk of insertional mutagenesis and genotoxicity, because the therapeutic gene does not disrupt endogenous genes [304,305]; a reduced potential of immune reactions compared to viral vectors [295]; and a large cloning capacity to accommodate therapeutic genes and regulatory elements [305]. Several types of episomal vectors have been developed, such as scaffold/matrix attachment region (S/MAR) episomes, which use genomic sequences for nuclear retention and segregation during mitosis [304,305]. In addition, minicircle episomes lack a bacterial backbone for reduced silencing [305]. Nano-S/MAR vectors with improved establishment and reduced cytotoxicity have also been developed [305]. While episomal vectors avoid the risks of genomic integration, they face challenges such as potential transgene silencing by epigenetic modifications and the loss of the episome over multiple cell divisions [304,305]. Ongoing research aims to improve their mitotic stability and expression for clinical applications.

CRISPR/Cas9 is a revolutionary tool adapted from a naturally occurring bacterial immune system. It has immense potential for treating genetic diseases by correcting disease-causing mutations and in the development of more effective cell therapies [306]. However, improving the technology’s precision, efficiency, and delivery methods remains a key challenge for clinical applications.

**Figure 3:**
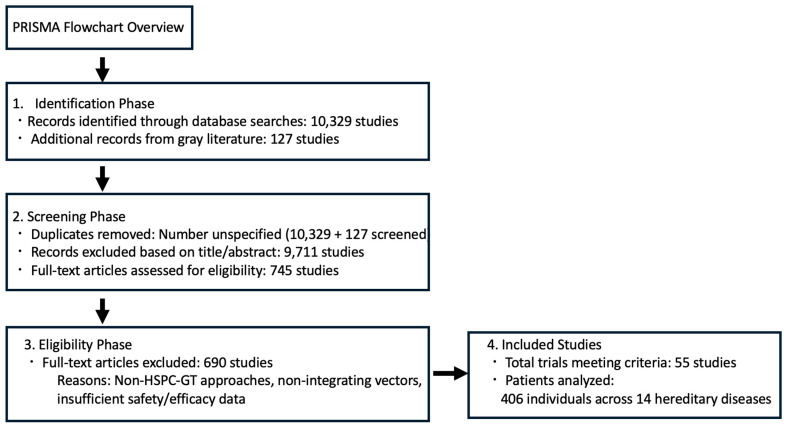
A PRISMA flowchart of study selection for gene therapy in hereditary diseases based on hematopoietic stem/progenitor cell gene therapy (HSPC-GT) trials from 1995 to 2020.

A PRISMA (Preferred Reporting Items for Systematic Reviews and Meta-Analyses) flowchart for gene therapy in hereditary diseases can be constructed based on a systematic review of hematopoietic stem/progenitor cell gene therapy (HSPC-GT) trials from 1995 to 2020 (Figure 3) [307]. This flowchart highlights the rigorous selection process and safety/efficacy outcomes in hereditary disease gene therapy trials, emphasizing the superior safety profile of lentiviral vectors [307].

## 9. Immune Memory of Gene Therapy with Oncolytic Viruses and CAR-T Cells

Gene therapy using oncolytic viruses and CAR-T cells represents a promising frontier in cancer treatment, particularly for solid tumors [308]. This combination approach leverages the strengths of both therapies to enhance overall efficacy and overcome the limitations of each individual treatment. Oncolytic viruses can improve the efficacy of CAR-T cell therapy by increasing tumor penetrance [308]. This is crucial for solid tumors, which CAR-T cells often struggle to infiltrate effectively. In addition, oncolytic viruses induce the oncolysis and immunogenic killing of tumor cells, modulating specific cell death pathways such as apoptosis, autophagic cell death, necrosis, and necroptosis [309].

Immune memory plays a critical role in the combination therapy of CAR-T cells and oncolytic viruses, enhancing the durability and efficacy of the treatment against cancer [310]. Oncolytic viruses promote immunogenic cell death, releasing tumor-associated antigens and danger signals that activate dendritic cells and other antigen-presenting cells [311]. This process leads to the activation of cytotoxic T lymphocytes and the generation of systemic antitumor immune memory, which helps control both primary and metastatic tumors [308]. The combination therapy enables CAR-T cells to acquire an immune memory phenotype [312]. Thus, immune memory is pivotal in this combination therapy, providing durable protection against cancer by ensuring prolonged CAR-T cell activity and systemic antitumor immunity. This mechanism addresses key limitations in treating solid tumors and improves long-term patient outcomes.

Several strategies have been developed to improve CAR-T cell infiltration and efficacy in solid tumors, such as fibroblast activation protein (FAP) targeting, matrix degradation, and endosialin (CD248) targeting [313]. FAP is expressed on fibroblasts in many solid tumors [314]. FAP CAR-T cells have shown effective tumor suppression and resulted in decreased numbers of FAP-positive stromal cells in multiple tumor models [315]. Also, Heparinase (HPSE)-modified CAR-T cells can promote matrix degradation and T cell infiltration. Some CAR-T cells are designed to secrete matrix-degrading enzymes to disrupt physical barriers in solid tumors. Furthermore, endosialin-targeting CAR-T cells have shown potential in depleting endosialin + stromal cells in subcutaneous and orthotopic tumors [313].

In addition, combining CAR-T cells with PD-1 inhibitors has shown increased efficacy in preclinical and clinical settings [316]. PD-1 blockade can increase the memory phenotype, reduce exhaustion, and induce durable responses of CAR-T cells [317]. Also, blocking multiple checkpoints (e.g., PD-1/TIGIT) has shown synergistic antitumor effects [318]. CTLA-4 and LAG-3 blockade in CAR-T cells has demonstrated stronger antitumor activity in various experimental animal models [312]. Furthermore, CRISPR technology has been used to generate PD-1- and TCR-deficient CAR-T cells, showing promising results in clinical studies [319].

Also, engineering CAR-T cells to express or respond to specific cytokines and chemokines can improve their infiltration and survival in the tumor microenvironment [119,316]. Knocking out TGF-βRII or incorporating a dominant-negative TGF-β receptor in CAR-T cells can confer resistance to the immunosuppressive effects of TGF-β [320]. Regional or local CAR-T cell administration (e.g., intratumoral, intraventricular, or intraperitoneal) has shown improved antitumor activity compared to intravenous delivery in some preclinical studies [321]. Thus, these strategies aim to overcome the unique challenges posed by solid tumors, including finding, entering, and surviving in the tumor microenvironment. Combining multiple approaches may lead to more effective CAR-T cell therapies for solid tumors.

## 10. Issues of Clinical Trials, Setbacks, and Regulatory Hurdles in Gene Therapy with Oncolytic Viruses and CAR-T Cells

### 10.1. Efficacy and Safety Concerns

Gene therapy using oncolytic viruses and CAR-T cells has shown great promise in treating various cancers, but several critical challenges have led to failed clinical trials, setbacks, and regulatory hurdles [77]. A primary reason for clinical trial failure is the inability to demonstrate efficacy [322]. This can occur due to flawed study design, inappropriate statistical endpoints, underpowered clinical trials (insufficient sample size), patient dropout, and insufficient enrollment. In addition, severe side effects have led to regulatory scrutiny and trial suspensions [275]. CAR-T cells can release large amounts of cytokines, causing high fever, trouble breathing, and other serious symptoms (cytokine release syndrome) [323]. Also, immune effector cell-associated neurotoxicity syndrome (ICANS) can result in confusion, seizures, and other nervous system problems [196]. In some cases, gene therapy trials have been suspended due to severe adverse events, including patient deaths [324].

### 10.2. Manufacturing and Delivery Challenges

CAR-T cell therapy involves a complicated manufacturing process that can lead to delays in treatment delivery, quality control issues, and high production costs [325]. The specialized nature of CAR-T therapy restricts its availability [119]. The treatment is typically limited to certified medical centers [326]. Patients often face long wait times, with a median of 6 months reported in one study. Geographic barriers may prevent eligible patients from accessing treatment centers.

### 10.3. Biological Challenges

Solid tumors present unique challenges for CAR-T cells, including restricted trafficking and infiltration into the tumor site and hypoxic and immunosuppressive conditions within tumors [145]. Cancer cells can evade CAR-T cell therapy by downregulating or losing the target antigen and exhibiting heterogeneous antigen expression [327]. Prolonged activation can lead to T cell exhaustion, reducing therapeutic efficacy [145].

### 10.4. Regulatory and Ethical Concerns

Regulatory bodies have suspended trials due to inadequate staff training and a lack of proper operating procedures [324]. The use of genetic modification raises ethical questions that can slow approval processes [328]. The expensive nature of CAR-T therapies can limit access. Treatment costs can range from EUR 50,000 to several hundred thousand euros [329]. Reimbursement restrictions can prevent or delay access to CAR-T therapies [326].

To address these challenges, researchers are exploring improved CAR designs to enhance safety and efficacy, strategies to overcome the immunosuppressive tumor microenvironment, more efficient manufacturing processes to reduce costs and wait times, and biomarkers to predict therapy outcomes and identify potential non-responders early [145,329]. By addressing these critical issues, the field of gene therapy with oncolytic viruses and CAR-T cells can potentially overcome current limitations and expand its applicability to a wider range of cancers [330].

## 11. Mechanistic Pathway of OVs Altering the TME Through Cytokine Signaling

Oncolytic viruses alter the TME through a complex mechanistic pathway involving direct tumor cell lysis and immune system modulation [331]. Oncolytic viruses selectively infect and replicate within tumor cells, leading to their lysis [77,332]. This process releases progeny virus particles, tumor-associated antigens (TAAs), damage-associated molecular patterns (DAMPs), and pathogen-associated molecular patterns (PAMPs). The release of these components triggers a cascade of immune responses, such as the recruitment of innate immune cells (e.g., NK cells, macrophages), activation of APCs, particularly DCs, and maturation and migration of DCs to draining lymph nodes [332,333]. Oncolytic viruses induce the production and release of various cytokines, which play a crucial role in modulating the TME. Pro-inflammatory cytokines attract and activate immune cells [81]. Interferon (IFN) response initially limits virus replication but also promotes antitumor immunity [334]. IL-2, IL-4, IL-12, IL-15, and IL-21 promote Th1-Tc1 antitumor immunity [333,335]. GM-CSF enhances DC migration and maturation and triggers systemic T cell responses [107]. Cytokine signaling and APC activation lead to the cross-presentation of TAAs to T cells in lymph nodes, expansion of tumor-specific CD4+ and CD8+ T cells, and infiltration of cytotoxic T lymphocytes (CTLs) into the tumor [332,333]. The combined effects of oncolytic virus infection and immune activation result in the transformation of “cold” tumors into “hot” immunogenic ones, the increased infiltration of effector immune cells, a reduction in immunosuppressive cells (e.g., Tregs, MDSCs), a shift in macrophage phenotype from M2 (immunosuppressive) to M1 (antitumor), and the potential disruption of tumor vasculature, facilitating immune cell migration [80,81]. The remodeled TME supports the ongoing destruction of tumor cells by both oncolytic viruses and immune cells, the development of immunologic memory, potentially reducing recurrence rates, and systemic antitumor effects, including responses against distant metastases [332,333]. By engineering OVs to express additional immunostimulatory molecules or combining them with other immunotherapies, practitioners can further improve their ability to remodel the TME and enhance antitumor immunity [77].

## 12. Conclusions

Viral vectors with high delivery efficiency are mainly used for gene transfer. In addition, oncolytic virus therapy, which uses a virus that selectively replicates and destroys cancer cells, is also considered a type of gene therapy because it uses recombinant viruses such as gene therapy vectors. Oncolysis releases new viruses and cancer antigens that not only lyse surrounding cancer cells but also remodel the TME and initiate an inflammatory cascade that stimulates the production of chemokines and cytokines. Activated cytotoxic T cells stimulated by these cells then infiltrate the tumor and destroy cancer cells. After CD19 CAR-T cell therapy, normal B lymphocytes also disappear, resulting in low levels of immunoglobulins that require replacement, known as on-target off-tumor responses. The TME remodeling induced by oncolytic virus infection improves the infiltration, persistence, and functional limitations of CAR-T cells, resulting in higher therapeutic efficacy. In addition, gene therapy technology can be applied not only to rare diseases but also to various other diseases.

## Figures and Tables

**Table 1:** Clinical trials using lytic viruses.

Virus	Name	Phase	Tumor	Reference
Adenovirus	ONYX-015	III	Squamous cell carcinoma head and neck (SCCHN)	[25]
		I/II	Pancreatic cancer	[26]
		pilot	Advanced cancers	[27]
		I/II	Advanced sarcoma	[28]
	Oncorine	III	SCCHN or esophageal cancer	[29]
	AD5-CD/Tkrep	I	Prostate cancer	[30]
	ONCOS-201	I	Solid tumors	[31]
Herpes simplex virus	Talimogene laherparepvec	I/II	SCCHN	[32]
		Ib	Melanoma	[33]
	RT3D	I	Glioma	[34]
Reovirus		I/II	Advanced cancers	[35]
